# Newly identified nematodes from the Great Salt Lake are associated with microbialites and specially adapted to hypersaline conditions

**DOI:** 10.1098/rspb.2023.2653

**Published:** 2024-03-13

**Authors:** Julie Jung, Tobias Loschko, Shelley Reich, Maxim Rassoul-Agha, Michael S. Werner

**Affiliations:** ^1^ School of Biological Sciences, University of Utah, Salt Lake City, UT, USA; ^2^ Max Planck Institute for Biology, Tübingen, Germany

**Keywords:** extremophile, hypersaline, phylogenetics, lacustrine sediment, nematodes

## Abstract

Extreme environments enable the study of simplified food-webs and serve as models for evolutionary bottlenecks and early Earth ecology. We investigated the biodiversity of invertebrate meiofauna in the benthic zone of the Great Salt Lake (GSL), Utah, USA, one of the most hypersaline lake systems in the world. The hypersaline bays within the GSL are currently thought to support only two multicellular animals: brine fly larvae and brine shrimp. Here, we report the presence, habitat, and microbial interactions of novel free-living nematodes. Nematode diversity drops dramatically along a salinity gradient from a freshwater river into the south arm of the lake. In Gilbert Bay, nematodes primarily inhabit reef-like organosedimentary structures built by bacteria called microbialites. These structures likely provide a protective barrier to UV and aridity, and bacterial associations within them may support life in hypersaline environments. Notably, sampling from Owens Lake, another terminal lake in the Great Basin that lacks microbialites, did not recover nematodes from similar salinities. Phylogenetic divergence suggests that GSL nematodes represent previously undescribed members of the family Monhysteridae—one of the dominant fauna of the abyssal zone and deep-sea hydrothermal vents. These findings update our understanding of halophile ecosystems and the habitable limit of animals.

## 1. Introduction

The Great Salt Lake (GSL) in northern Utah fluctuates around 15% salinity in its southern arm and 30% in its northern arm, making it one the most saline bodies of water on Earth. Early non-indigenous settlers assumed it was too saline to harbour life [1], but modern methods have revealed a thriving extremophile community inhabiting the lake's benthic zone [2–4]. The GSL's shallow marginal areas are home to salt-tolerant microorganisms that build calcium carbonate mounds approximately 1 m in diameter called microbialites [2,3,5]. These reef-like structures span 700–1000 km^2^, or approximately 20% of the lake bottom, making it one of the largest assemblages of microbialites in the world. While rare today, microbialites were the dominant macroscopic evidence of life on Earth for two billion years [6]. Understanding the formation and biota of these structures could provide important clues to the origin and ecology of early life on our planet.

Despite the lake's flourishing microbial community, only two metazoan taxa have persisted in the hypersaline waters of Gilbert Bay, brine shrimp (*Artemia* sp.) and brine fly larvae (*Ephydra* sp.), as well as single celled protozoans [7]. These invertebrates are a critical food source for millions of migratory birds passing through the Pacific and Central flyways annually [2,8]. Yet, like many of the world's saline lakes, the GSL is shrinking at an alarming rate [9]. Decades of water diversion exacerbated by severe drought in the region have caused historic lows in water elevation [10]. As a result, exposed microbialite habitat and record-high salinity levels threaten both benthic zone inhabitants and the upper trophic levels that depend on them. Thus, there is a pressing need to understand this lynchpin community and the limits of their habitability.

Nematodes are a nearly ubiquitous and diverse meiofaunal taxa. Although free-living nematodes have not been described in the GSL, several lines of evidence motivated further investigation [11]. As the most abundant animal phylum on the ocean floor and terrestrial biosphere, nematodes exhibit remarkable diversity with an estimated 250 000 species and at least 25 000 known extant species [12–15]. Some of these have been found kilometres below the surface [16], and in extreme cold and arid conditions [17]. Moreover, a recent study reported several nematode species in Mono Lake, an arsenic-rich terminal lake in California [18,19].

Here, we report the recovery of nematodes from the benthic zone of the GSL—the most saline environment from which nematodes have been described [20]—and define their preferred habitat, microbiome and phylogenetic relationships. These findings raise the number of metazoan taxa in Gilbert Bay's benthos from two to three, extend the known habitable zone of nematodes, and provide a foundation for future studies of extremophile animal–microbe communities.

## 2. Halophilic nematodes live in the Great Salt Lake

**Figure 1.**
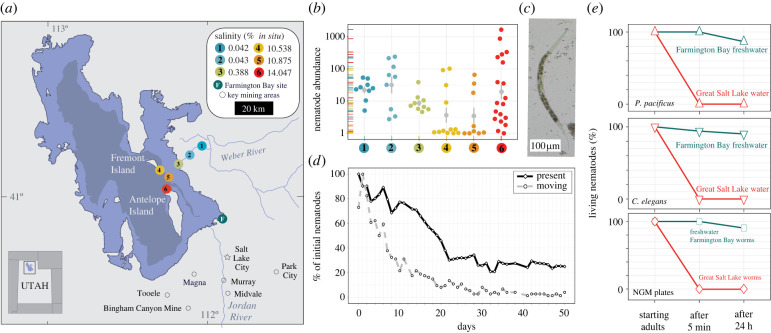
Nematodes live in the Great Salt Lake, Utah. (*a*) Locations and average *in situ* water salinity of the six sampling sites around (1–3) and within (4–6) the Great Salt Lake (GSL). Lake topographies depict NASA SRTM2 v.2 data from 2007 and UGRC LiDAR data from 2016. Samples were collected in spring, summer, and autumn of 2021 and the summer of 2022. (*b*) Nematode abundance measured in number of nematodes per 100 g of dry lake sediment on a log scale and coloured by sample site from which samples were collected, spread horizontally to show stacked points. Vertical grey lines show mean and standard error. (*c*) Representative nematode extracted from the south arm of the GSL. (*d*) Motility (grey circles) and presence (black diamonds) of GSL nematodes (*N* = 268) when placed in the 14% salinity water from which they were recovered. (*e*) Survival of *C. elegans* and *P. pacificus* before and after being placed in hypersaline GSL water (salinity 14%) and Farmington Bay freshwater (salinity 0.01%) for 5 min, and survival of nematodes extracted from Farmington Bay and the GSL before and after being placed on standard nematode growth media (NGM) plates for 5 minutes.

We investigated the biodiversity of meiofauna in the GSL by conducting seasonal sampling from the spring of 2021 to the summer of 2022. Our field sites spanned a salinity gradient that started in the freshwater Weber River (sites 1 and 2), transitioned to a brackish delta (site 3), and terminated in three sites within the south arm of the GSL (sites 4–6; figure 1*a*; electronic supplementary material, figure S1).

We began by collecting submerged sediment samples and using the standard Baermann funnel technique to extract nematodes [21], but did not recover any live worms from saline sites over several sampling trips. However, when we used a sucrose-density centrifugation method, which can process larger samples and has historically been used to isolate meiofauna from low density sites in desert and polar regions [17], we consistently recovered brine fly larvae, brine fly cysts and nauplii and live nematodes (figure 1*b*,*c*; electronic supplementary material, movie S1). To confirm whether GSL nematodes were true halophiles, we compared their viability in GSL water to that of two common laboratory nematodes: *Caenorhabditis elegans* (N2) and *Pristionchus pacificus* (PS312). GSL nematodes' motility and survival declined steadily for the first three weeks and then plateaued (figure 1*d*). After 21 days, 27.7% of initial nematodes remained present—some possibly in a suspended state as seen in several extremophiles [22]—and 4.7% remained motile at least for the following 29 days (figure 1*d*). By contrast, all individuals of *C. elegans* and *P. pacificus* ruptured and/or died within 5 min of being placed in GSL water (figure 1*e*). These strains typically have an average lifespan of 19 and 22 days, respectively [23,24]. Importantly, the vast majority of *C. elegans* (94.1%) and *P. pacificus* (100%) survived being placed in freshwater (salinity 0.01%) collected from a wildlife management area in Farmington Bay (40°54′24.7″ N, 111°55′18.1″ W) (figure 1*a*,*e*). Similarly, 100% of nematodes isolated from Farmington Bay freshwater sediment survived 5 min on standard nematode growth media (NGM) plates [25] (figure 1*e*). However, 100% of GSL nematodes were immobile and appeared dead within five minutes when placed on NGM plates (figure 1*e*), indicating specific adaptation to the environment of the GSL.

We attempted several methods to culture GSL nematodes (electronic supplementary material, table S1), but so far have not found conditions that show evidence of reproduction. Interestingly, this is in contrast to nematodes recovered from Mono Lake and deep underground mines, which were culturable in relatively standard conditions [16,18]. We conclude that GSL nematodes are true halophiles.

**Figure 2.**
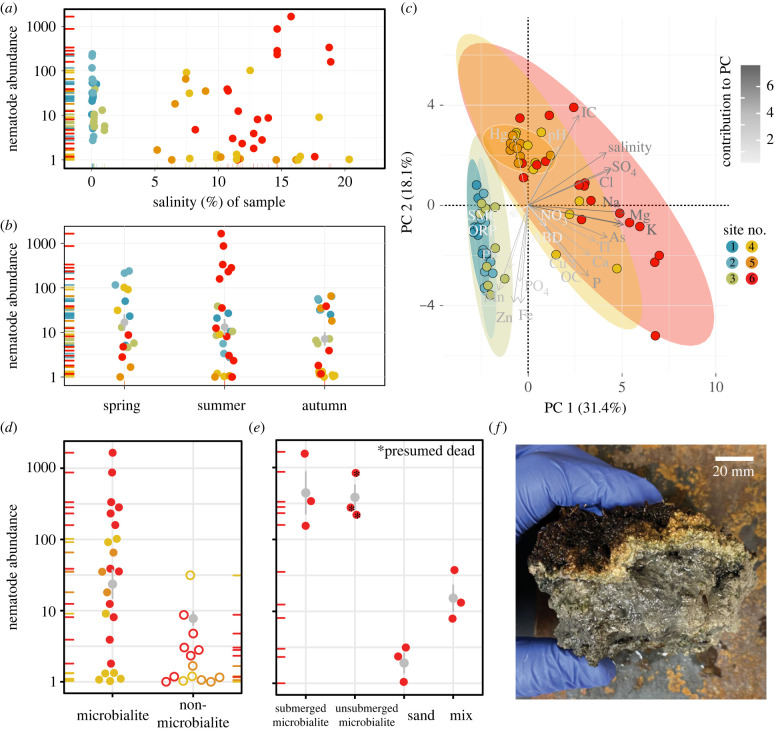
Microbialites are a microhabitat for GSL nematodes. Nematode abundance by (*a*) salinity of each sample and (*b*) season during which each sample was collected. (*c*) Principal component (PC) analysis of samples by site. Colour and length of line in biplots are determined by contribution to the PC. Ellipses show 95% confidence intervals. ABD: abundance, SMC: soil moisture content, ORP: oxidation–reduction potential, IC: inorganic carbon, OC: organic carbon. (*d*) Nematodes are preferentially found in microbialite (filled) compared to non-microbialite (unfilled) samples from GSL. (*e*) Nematode abundance, as collected from submerged microbialite, recently unsubmerged microbialite, submerged sand directly adjacent to microbialites, and a microbialite/sand mix. Those marked with black stars indicate all extracted nematodes were dead upon extraction. (*f*) Representative microbialite sample. Abundance was measured in number of nematodes per 100 g of dry lake sediment on a log scale. Data points for categorical variables are individual samples coloured by sample site from which samples were collected and spread horizontally to show stacked points. Vertical grey lines show mean and standard error.

Next, we assessed potential correlates of nematode abundance with environmental factors. Average nematode abundance in the GSL (median 7 per 100 g dry sediment) was lower than typically found in terrestrial ecosystems (median range: 81 to 2329 per 100 g dry sediment), but similar to the densities found in Mono Lake (median 6 per 100 g dry sediment) (electronic supplementary material, table S2) [13,18]. Surprisingly, nematode abundances in hypersaline GSL sites were comparable to densities in nearby freshwater Weber River sites (Sum Sq = 2.056, d.f. = 1, *f* = 3.05, *p* = 0.09, ANOVA). Consistent with this observation, salinity did not drive differences in nematode abundance across sample sites (Sum Sq = 0.0151, d.f. = 1, *f* = 0.028, *p* = 0.868, ANCOVA; figure 2*a*). While the benthic ecosystem of the GSL is adapted to hypersaline conditions, salinity above 15% has been shown to negatively affect the microbial mat community and brine shrimp and brine fly growth [26–28]. Interestingly, we found relatively high nematode abundances in samples of up to 19% salinity. Abundances also did not change from spring through the autumn (Sum Sq = 1.531, d.f. = 2, *f* = 1.105, *p* = 0.338, ANOVA; figure 2*b*), which suggests these animals can tolerate large shifts in UV, temperature, and salinity that accompany the seasonal changes in high desert environments [29,30].

Nevertheless, additional sampling in the summer of 2022 yielded significantly fewer nematodes concurrent with a record low lake elevation and mean annual loss in elevation of 35.76 cm (mean difference in abundance: 72.64 nematodes per 100 g dry sediment; *Z* = 3.16, *p* = 0.00080, Wilcoxon rank sum test). Presumably this was due to the cumulative effect of multiple years of exposure and higher than normal salinity on nematode physiology, their bacterial food or both. Thus, while these newly discovered inhabitants of the GSL benthos are remarkably resilient, their ability to tolerate extreme lows in lake elevation and/or high salinity has its limits.

## 3. Microbialites are a habitat for Great Salt Lake nematodes

In the absence of a correlation of abundance with salinity, we searched for other covariates of nematode habitability. We measured pH, soil moisture content, oxidation–reduction potential, soluble anions and extractable elements (Na, Mg, K, Ca, P, Mn, Fe, Cu, Zn, As, Hg, Tl and Pb), and total organic carbon and inorganic carbon. Sites within the GSL had a distinct chemical profile as compared to our freshwater samples (figure 2*c*). More than a third (35%) of GSL samples exceeded the threshold effect concentration of arsenic (10 µg g^−1^), which is the concentration above which adverse effects on sediment-dwelling organisms can occur, and the highest exceeded the limit by 1100% [31]. Surprisingly, we observed a marginal positive correlation of arsenic and nematode abundance. However, beyond arsenic, we did not observe a significant effect of any element or compound on nematode abundance (electronic supplementary material, figure S2 and table S3).

The lack of a strong signal from abiotic environmental sources motivated us to search for biotic correlates of nematode abundance. Through our sampling efforts, we noticed higher numbers of nematodes from microbialite sediment than from adjacent non-microbialite sediments (*Z* = 3.55, *p* = 0.0002, Mann–Whitney Wilcoxon rank sum test; figure 2*d*). To further investigate this association, we homogenized microbialite samples and passed the crushed sediment through size-selective sieves. Crushed microbialite material yielded on average 237 times more living nematodes than did sediment directly adjacent to microbialite mounds. Meanwhile, a mix of microbialite and non-microbialite sediment exhibited intermediate nematode abundances (Sum Sq = 11.864, d.f. = 3, *f* = 29.424, *p* = 0.0001134, ANOVA; figure 2*e*). Thus, GSL nematodes are associated with microbialites.

Microbialites could provide a reliable source of bacterial food for GSL nematodes. Indeed, the nematodes identified from our sampling were likely bacterivores based on their relatively narrow and smoothly lined buccal cavities (electronic supplementary material, figure S3). Additionally, microbialites may also provide a physical shelter from harsh environmental conditions. Most of the microbialites sampled in this study subscribed to previously described morphology, each showing a succession of thin orange and porous green layers (figure 2*f*) [26]. The top layer exhibited clusters of photosynthetically active coccoid cyanobacteria and carotenoids, which quenches UV rays and protects the deeper layers [5]. We also extracted nematodes from dry exposed microbialites; however, they were immobile, rigid, and presumed dead (figure 2*e*). Therefore, the protective and/or nourishing habitat of microbialites likely depends on their submergence.

## 4. The microbiome of Great Salt Lake nematodes

Both endo- and ecto-symbiotic (i.e. cuticular) bacteria are capable of providing niche-specific functions for their nematode hosts [32–35]. To identify sources of food and potential symbiotic relationships within microbialites we sequenced the V4 region of bacterial 16S rRNA from 15 individual nematodes from site 4. The median Shannon alpha diversity of bacteria across nematodes was 4.8 (range 4.5 to 5.3) and the median diversity among archaea was 2.2 (electronic supplementary material, figure S4), demonstrating a relatively rich microbial community. Previous studies on lakes of the Qinghai–Tibetan Plateau, China, and the Monegros Desert, Spain, have identified comparable microbial diversity with the Shannon diversity index ranging from 3.3 to 6.4 and 1.5 to 2.2, respectively [36,37]. Importantly, rarefaction curves indicated that our analysis captured all the diversity of the true microbial community (electronic supplementary material, figure S5). Moreover, our ‘no nematode’ control samples did not exhibit PCR amplification, arguing that the associated microbial taxa resided either on or inside the worms (electronic supplementary material, figure S6).

**Figure 3.**
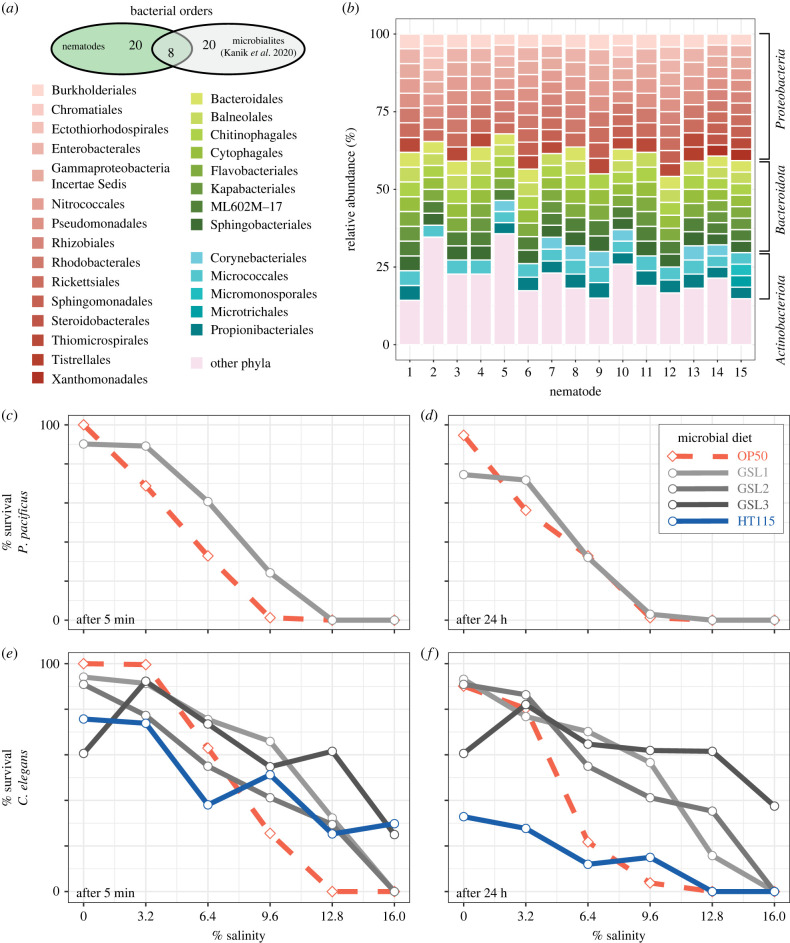
Microbiome of GSL nematodes and their potential for improving nematode survival. (*a*) Venn diagram of bacterial orders specific to GSL nematodes, specific to GSL microbialites [3], and shared between the two. (*b*) Overview of nematode-associated microbes. The relative abundance of nematode-associated bacterial communities binned by order. All 15 nematodes were collected from site 4 and taxonomic bins are grouped by phylum. (*c,d*) Percent survival of *P. pacificus* and (*e,f*) *C. elegans* reared on standard NGM plates spotted with the standard *E. coli* OP50 strain (red) versus GSL microbes cultured from microbialite chunks (greys) 5 min and 24 h post-submergence in a dilution series of 16% saline GSL water. Each point is an average of all tested nematodes at the indicated salinity (*P. pacificus*: N0% = 158, N20% = 140, N40% = 104, N60% = 193, N80% = 194, N100% = 150; *C. elegans*: 359, 688, 501, 452, 493, 332).

Photosynthetic bacteria are typically dominant in the surface layers of microbialites whereas chemo-heterotrophs and anaerobes are dominant in the deeper layers [5,38,39]. Only 4% of amplicon sequence variants (ASVs) associated with GSL nematodes mapped to cyanobacteria, suggesting nematodes are associated with the deeper, more protected layers of microbialite. Several of the remaining bacterial orders overlapped with orders found in microbialites sampled from the south arm of the GSL (figure 3*a*) [3]. For example, Proteobacteria were one of the dominant phyla found to be associated with GSL nematodes and are known to deposit carbonate by sulfate reduction for microbialite formation [29]. Surprisingly however, approximately 70% (20/28) of bacterial orders were specific to nematodes [3] (figure 3*a*). Thus, single-worm 16S-sequencing revealed a rich and largely distinct nematode–microbial community (figure 3*b*).

The dominant taxa associated with GSL nematodes ­were *Psychroflexus* and *Candidatus aquiluna*, which belong to the most abundant bacterial orders Flavobacteriales and Micrococcales, respectively (electronic supplementary material, figure S7*a*). These are known to act as secondary producers by supplementing their energy metabolism using light-harvesting rhodopsin pigments and other photosynthetic reaction centres [3,29]. Similarly, one of the eight bacterial orders shared between nematodes and microbialites is Chromatiales, which are known anoxygenic photosynthesizers [40]. GSL nematodes are also associated with the halotolerant bacterium *Halomonas elongata* (electronic supplementary material, figure S7*a*) and several halophilic archaea that are known to regulate the amount of salt and osmotic pressure inside their cells by keeping charged solutes and amino acids on their cell surfaces (electronic supplementary material, figure S7*b*) [41–44]. Interestingly, members of the dominant archaeal phylum present in GSL nematodes, Halobacteriota, can use arsenic for bioenergetic processes [45,46], which may be related to the positive association of arsenic and nematode abundance (electronic supplementary material, table S3) [47]. Different oxidized forms of arsenic can serve as electron donors in anoxygenic photosynthesis [48] or terminal acceptors in anaerobic respiration by chemoautotrophic bacteria [49]. While it is unknown whether the microbes in the GSL use arsenic for metabolism, it appears to be widespread among microbialite bacteria in comparable hypersaline lakes in the Andes [50].

Taken together, the GSL nematode microbiome contains several members with known mechanisms of carbon fixation, hypersaline tolerance and toxic-chemical resistance. These associations may reflect ancient animal–microbe interactions within microbialites, and are attractive candidates to investigate habitat-adapted symbiosis or coevolution to the extreme environment of the GSL.

## 5. Great Salt Lake microbes increase nematode survival to high salinity

Without being able to culture GSL nematodes we could not directly test whether microbialite bacteria are required for tolerance to hypersaline conditions. However, to assess the sufficiency of GSL bacteria for salinity tolerance, we reared the model laboratory nematodes *C. elegans* and *P. pacificus* on microbes cultured from GSL microbialites. Then, we exposed adult worms to five minutes of dilutions of GSL water, which was 16.0% salinity at the time of the experiment (figure 3*c–f*). For each tested individual, we measured survival five minutes and 24 h after the salinity stress. Growth on GSL bacteria significantly increased survival after five minutes for both species across multiple salinities compared to the laboratory strain of *Escherichia coli* (OP50) (*P. pacificus*, *N* = 601: Chi Sq = 16.16, d.f. = 1, *p* = 5.809 × 10^−5^, ANOVA, figure 3*c*; *C. elegans*, *N* = 911: Chi Sq = 111.70, d.f. = 1, *p* < 2.2 × 10^−16^, ANOVA, figure 3*e*). *C. elegans* generally survived better than *P. pacificus* in response to salinity stress (5 min: Chi Sq = 138.54, d.f. = 1, *p* < 2.2 × 10^−16^, ANOVA; 24 h: Chi Sq = 71.85, d.f. = 1, *p* < 2.2 × 10^−16^, ANOVA), but that effect depended on microbial diet at 24 h (Chi Sq = 19.69, d.f. = 1, *p* = 9.11 × 10^−06^, ANOVA). A diet of GSL microbes had a diminished effect on *P. pacificus* at 24 h (Chi Sq = 0.13, d.f. = 1, *p* = 0.717672, ANOVA; figure 3*d*). However, *C. elegans* continued to have significantly higher survival (Chi Sq = 125.63, d.f. = 1, *p* < 2.2 × 10^−26^, ANOVA; figure 3*f*). Remarkably, several living *C. elegans* animals were recovered 24 h following exposure to 16% salinity GSL water, which is more than 50 times the salinity of their standard growth conditions (figure 3*f*).

We were curious if the increased survivability on GSL microbes was due to a general induction of stress response to a foreign bacterium. *E. coli* HT115 is a weak biotic stressor that, perhaps counterintuitively, can enhance survival of *C. elegans* under certain stressed conditions [51]. The laboratory strain of *C. elegans* (N2) fed on HT115 showed a mixed response to GSL water after 5 min (ANOVA, diet: Chi Sq = 0.0131, d.f. = 1, *p* = 0.2025; diet × salinity: Chi Sq = 187.42, d.f. = 1, *p* < 2 × 10^−16^; figure 3*e*). However, after 24 h, the HT115 diet reduced survival in *C. elegans* rather than improving it (ANOVA, Chi Sq = 40.63, d.f. = 1, *p* = 1.84 × 10^−10^), especially at low salinities (ANOVA, Chi Sq = 75.83, d.f. = 1, *p* < 2.2 × 10^−16^; figure 3*f*). Thus, GSL microbes can increase salinity tolerance of laboratory nematodes, and this effect appears to be specific to these microorganisms.

## 6. Genetic diversity of Great Salt Lake nematodes

**Figure 4.**
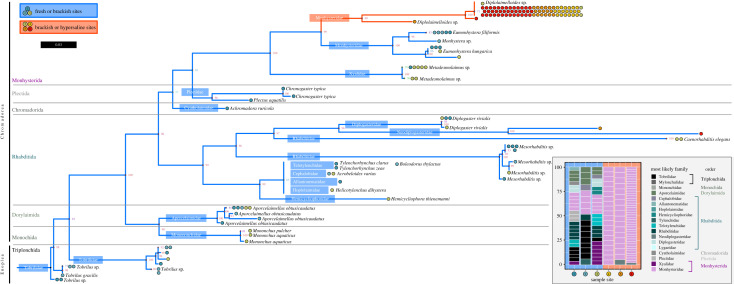
Diversity of Great Salt Lake nematodes. Maximum-likelihood phylogenetic tree of isolated nematodes, based on aligned SSU sequences. Tips denote the best BLAST hit (NCBI) greater than 97% identity and are coloured by the site from which the nematode was collected. Nodal support was performed with 1000 bootstrap replicates. (Inset) Nematode family composition by site. Each box is an individual nematode, coloured by the most likely family and order.

Next, we performed small subunit ribosomal RNA (SSU) sequencing to genotype nematodes from each site and thereby facilitate their identification (electronic supplementary material, table S4) [52]. Nematodes found in our freshwater and mostly freshwater sites (1–3) belonged to 18 different families. However, all but two nematodes extracted from the south arm of the GSL belonged to a single family: Monhysteridae (figure 4). With more than 200 species and global distribution across marine and estuary sediment, Monhysteridae are likely an ancient nematode family [53,54]. Monhysterids have been found miles below the surface in South African mines [16], as well as in Sivash Bay [55], Mono Lake [18] and the Aral Sea [56]. Moreover, Monhysteridae is one of the most abundant nematode families in deep-sea hydrothermal vents and its members are often the dominant metazoan in abyssal zone sediment [57], demonstrating an ability to adapt to extreme environments. The dramatic drop in diversity in sites 4–6 corresponds with the transition from brackish delta to hypersaline sites in the lake, indicating adaptation of specific lineages to extreme salinity.

To investigate the evolutionary history of GSL nematodes we constructed a maximum-likelihood phylogenetic tree based on single-worm SSU sequences. Our tree recapitulated established phylogeny [52,58], and included two of the three major lineages that exist within phylum Nematoda, class Enoplea and class Chromadorea (figure 4). Several primer pairs that are used to genotype other loci failed to amplify DNA of nematodes from Gilbert Bay (electronic supplementary material, table S4), consistent with a large degree of divergence. However, a tree made from a second 18S locus exhibited the same overall topology (electronic supplementary material, figure S8), providing confidence in the phylogenetic relationship between nematodes found in Gilbert Bay and adjacent freshwater sites.

Using a conservative bootstrap threshold of 95%, we observed two well-defined clades within Monhysteridae. All 80 nematodes in the first clade were collected from brackish and hypersaline sites (sites 3–6), and the vast majority (greater than 97%) were from hypersaline sites 4–6. By contrast, all 11 nematodes in the second clade were collected from freshwater and brackish sites (sites 1–3) (figure 4). Monhysterids from the GSL also differed morphologically from Monhysteridae worms from our freshwater samples (electronic supplementary material, figure S4). Freshwater Monhysteridae from sites 1–2 were larger (electronic supplementary material, figure S3*a*) and displayed two conspicuous cephalic setae (electronic supplementary material, figure S3*b*,*c*), which were missing or substantially smaller in GSL monhysterids (electronic supplementary material, figure S3*d*–*f*) [59]. Three specimens in the hypersaline clades appear to be from the genus *Diplolaimella*, a free-living group that typically inhabits marine and coastal sediment. However, morphological descriptions (i.e. alpha taxonomy) coupled with transcriptomic and genomic sequencing are on-going to reveal the precise number of GSL species and their phylogenetic position. Notably though, the vast majority of nematodes from sites 4–6 exhibited less than 97% best-hit match on NCBI. The combination of (1) adaptation to extreme salinity, (2) large sequence divergence and (3) evidence of distinct branches suggests a prolonged period of reproductive isolation. We interpret these data as at least one, if not several, novel species of nematodes unique to the south arm of the GSL.

## 7. Comparison to an analogous terminal saline lake in the Great Basin

**Figure 5.**
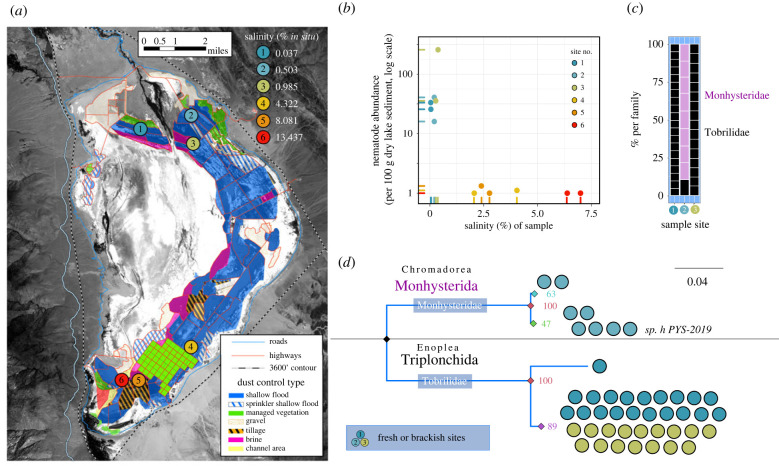
Comparison of nematodes isolated from the restored Owens Lake, CA. (*a*) Locations and *in situ* salinities of the six sampling sites. Original map credit: LADWP. (*b*) Nematode abundance by salinity of each sample. (*c*) Nematode family composition by site. Each box is an individual nematode, coloured by the most likely family. (*d*) Maximum-likelihood phylogenetic tree of isolated nematodes, based on aligned SSU sequences. Tips denote the best BLAST hit (NCBI) greater than 97% identity and are coloured by the site from which the nematode was collected. Nodal support was performed with 1000 bootstrap replicates.

To investigate the potential evolutionary history of GSL nematodes, we collected submerged benthic sediment from six sites in Owens Lake, a terminal lake on the western edge of the Great Basin that does not have microbialite structures (figure 5*a*). Once one of the largest inland bodies of water in the USA, Owens Lake was drained by 1926 to accommodate the burgeoning water demand of Los Angeles. In 2006, the Los Angeles Department of Water & Power began an expansive effort to mitigate dust pollution, ranging from gravel cover and tilled sediment to managed vegetation and shallow flooding (figure 5*a*), generating sites with different salinities. We recovered 15–257 nematodes per 100 g of sediment from the three least saline sites, where measured salinity was less than 1% (figure 5*a*,*b*). However, the remaining sites—which ranged from 4.3% to 13.4% salinity *in situ* (2.05–7.00% in sediment)—yielded negligible nematode abundances (figure 5*b*). Thus, in contrast to the GSL, nematode abundance in Owens Lake was inversely correlated with salinity (figure 5*b*).

We also discovered notably less diversity at the freshwater sites within Owens Lake compared to freshwater input sites near the GSL. Nematodes from sites 1 and 3 were primarily in the family Tobrillidae, while nematodes from site 2 were primarily in the family Monhysteridae (figure 5*c*). Interestingly, the monhysterids found in site 2 seem to be the same SSU haplotype (sp. h PYS-2019) discovered in 2016 in Mono Lake, located just 140 miles northwest of our sites [18]. Given the similar geology between the GSL and Owens Lake, the large differences in nematode abundance and taxa were notable. We attribute these differences to two non-mutually exclusive possibilities: decades of desiccation caused a major local extinction event in Owens Lake, or the microbialite mounds within the GSL provided a protective habitat for nematodes to thrive in hypersaline environments. In either case, sampling data from Owens Lake suggest that the continued survival of nematodes in Gilbert Bay depends on the water level of the GSL.

## 8. Conclusion

Gilbert Bay in the GSL is an extreme hypersaline environment with only two currently known metazoan groups reliably found in its benthic zone: brine flies and brine shrimp. Here, we report the discovery of halophilic nematodes in association with microbialites. At up to 20% salinity, to the best of our knowledge this represents the most saline environment from which nematodes have been recovered. Phylogenetic analysis of recovered nematodes suggests that they represent at least one, if not multiple previously undescribed species. Collectively, our findings expand the biodiversity of the GSL and introduce a new system (i.e. nematodes, bacteria, microbialites) to study the ecology and evolution of extremophile animals in early-Earth analogue environments.

The major habitat of GSL nematodes appears to be microbialites—a critical source of primary production for the entire GSL ecosystem [26]. Nematodes likely benefit from grazing on the bacteria in microbialites and may also gain protection from UV exposure or dehydration [5]. It is currently unclear whether the relationship is reciprocally beneficial towards microbialites and their microbial communities, but nematodes are known to contribute to niche construction through grazing, nutrient cycling and bioturbation in diverse environments [12]. Going forward, a particular focus will be applied to whether these functions facilitate the accumulation of sediment layers in microbialites.

Surprisingly, the majority of identified microbes were distinct to their nematode hosts, indicating some may provide additional functions beyond food [32,33]. For example, *C. elegans* fed the *Lysinibacilus sphaericus* B1CDA had a longer lifespan on arsenic than *C. elegans* fed *E. coli* OP50 [60]. Similarly, chemosynthetic endosymbionts of the stilbonematid nematode *Laxus oneistus* actively fix nitrogen, providing essential nutrients to their hosts [34]. Our study suggests that microbes from the GSL can also provide salinity tolerance to a broad range of nematode hosts. Future studies will investigate the underlying mechanisms of this interaction and whether these relationships have facilitated survival in the extreme environment of the GSL. Nevertheless, the sharp decline of nematode diversity in hypersaline sites suggests that there are species-specific adaptations as well.

The discovery of nematodes in the GSL benthic zone presents several evolutionary questions, especially given historical fluctuations in the lake's size and salinity levels. For much of the last 800 000 years, our sampling sites have likely stood in a saline lake [61]. However, approximately 30 000 years ago the GSL was part of a vast freshwater lake that covered most of western Utah named Lake Bonneville. Thus, any long-term inhabitants of the GSL have had to face severe evolutionary pressures from repeated environmental change. Alternatively, current biota may have colonized the hypersaline niche more recently. Understanding the establishment and evolution of hypersaline benthic ecosystems—including colonization history and co-evolution with microbialites—could help us trace the boundaries of life on our changing planet, with implications for where complex life may be found elsewhere.

## 9. Material and methods

### (a) Sites and sampling

Sediment samples were collected from six sites of varying salinities in and around the GSL (figure 1*a*; electronic supplementary material, figure S1) in spring, summer and autumn of 2021 and summers of 2022 and 2023. On 26 July 2021, the southern arm of the GSL hit a historic low (4191.28 feet above sea level), surpassing the previous record set in 1963. We collected samples from site 6 four days prior to and eight days after that day. The sediment samples collected were taken from both microbialite and non-microbialite structures, allowing quantification of microbialite effects. Microbialite samples collected in summer and autumn were crushed with a hammer prior to extraction. In the summers of 2022 and 2023, we collected only from microbialites at site 6 and along the water's receding edge. At each site (within 50 m of the GPS point), at least two samples, or plastic buckets were filled with 400–600 ml of underwater sediment (less than 10 cm) using a shovel or PVC pipe cupped at one end by hand (mean *N* samples: 3.14; range: 2 to 5). We also collected two non-microbialite sediment samples from six sites of varying salinity within the restored Owens Lake on 16 June 2022 (figure 5*a*). *In situ* salinities at every site were measured with a YSI Professional Plus probe fitted with a conductivity meter. All proper permits for sampling sediment and water were acquired from the State of Utah (2021: 420–00334, 2022: 410–00741) and the Los Angeles Department of Water and Power (2022: 2203342).

### (b) Sediment measurements

All sediment samples were returned to the laboratory and processed over the following week. A total of 10 ml of sediment was diluted with deionized water 1 : 10 (100 ml final) and mixed with a spatula to homogeneity. Dilute samples were probed with a YSI Professional Plus probe fitted with a conductivity meter to measure salinity (ppt, converted to %) and oxidative–reductive potential (ORP). pH was also measured on the diluted sample using an Accument AE150 probe (Fisher Scientific). For measuring dry weight, 10 ml of sediment was weighed in an autoclaved glass beaker and dried for 48 h at greater than 100°C, then re-weighed. Soil moisture content was calculated as: (wet weight − dry weight)/dry weight. Abundance, or number of worms per 100 g of dry sediment, was calculated as: (worms counted/volume of sediment sample) × (volume of sediment weighed/wet weight) × (wet weight/dry weight) × 100. Approximately 1 g of dried sediment was analysed for environmental chemical analysis using inductively coupled plasma mass spectrometry (ICP-MS), ion chromatography mass spectrometry (IC-MS), and IC (see electronic supplementary material, methods).

### (c) Bacterial identification

Bacterial communities associated with individual nematodes were extracted from a single microbialite sample collected on 3 May 2021 from site 4, the southern tip of Fremont Island. We isolated 16 worms in 10 µl each of single worm lysis buffer (without Proteinase-K) and incubated in a thermocycler for 1–2 h at 60°C. Using 5 µl as input, we amplified the V4 region of the 16S rRNA gene using the region-specific primer pair 515F and 806R, which included adapter sequences used on the Illumina MiSeq flow cell [62]. As negative controls, we used 8 samples from picks dipped in site-specific water but without transporting a worm. These controls did not amplify in the gel, indicating that sequenced microbes were specific to their worm hosts (electronic supplementary material, figure S6).

After quantification, the pool was diluted to 4 nM (confirmed via Qubit), denatured, and then further diluted to a final concentration of 9 pM with a 10% PhiX. 16S rRNA gene amplicons were sequenced using multiplexed, paired-end 2 × 300 bp Illumina MiSeq sequencing. Cluster density was 486 K mm^−2^; clusters passing filter was 98.7%; % > Q3 was 93.1%; and estimated yield was 7319.3 MB with 6.63 × 10^6^ bacterial sequences. Post-sequence processing was performed with DADA2. The first 20 base pairs were truncated, as well as lower quality reads at the first instance of a Q score less than or equal to 2. Remaining forward reads were trimmed to 290 base pairs and reverse reads were trimmed to 250 base pairs. High-quality sequences were dereplicated and denoised, then paired-end reads were merged. Next, we filtered chimeric reads from our sequence table and assigned taxonomy using the Silva database for classification of 16S sequences. We assigned bacteria at the level of ASV. Taxa present in less than 1% relative abundance were removed from subsequent analysis. After filtering, our datasets still generated 6.04 × 10^6^ bacterial sequences from 15 individual nematodes.

### (d) Nematode identification

Nematodes were isolated from sediment samples by sucrose density centrifugation. Isolated nematodes were further identified by morphology and molecular signatures. Morphological observations were made on live specimens, anaesthetized using 20 mM sodium azide on 2% agarose slides under a Zeiss Axiolab 5 microscope fitted with an Axiocam 208 colour camera. For molecular analysis, individual worm lysate was prepared in single worm lysis solution. Two of 11 attempted universal or nematode-specific primer pairs were successful in amplifying DNA of the expected size, both of which targeted the 18S ribosomal small subunit (SSU rDNA) (electronic supplementary material, table S4). The gene fragments were amplified [12,18] and sequenced using standard PCR amplification. Samples with visible bands of the expected size and purity were selected for Sanger sequencing at the University of Utah DNA Sequencing Core Facility. From aligned SSU sequences, we constructed phylogenetic trees of isolated nematodes (figure 4; electronic supplementary material, figure S8). Nematode sequences were only included in the tree if their closest BLAST hit recovered greater than 80% query cover. Sequence alignment was performed using MAFFT version 7.490. Analysis was performed in IQ-TREE multicore version 2.1.3 using a general time reversible (GTR) model for nucleotide substitutions. Nodal support for maximum-likelihood analysis was performed for species identification with 1000 bootstrap replicates. If the isolated nematode exhibited greater than 97% identity to its best SSU BLAST hit (NCBI), that individual's tip got labelled with the relevant taxonomic information. Nodes with support values below 95 were collapsed into polytomies unless the predicted phylogeny recapitulated the known phylogeny of nematodes.

### (e) Survival assay

We extracted 268 nematodes from sediment collected at site 6 and recorded the number present and number actively moving immediately after extraction and every 24 h following being placed in GSL water collected from the lake on the same day as sample collection (= 14% salinity). We similarly recorded the viability of two common laboratory model nematodes in a dilution series of 16% salinity GSL water. We placed *Caenorhabditis elegans* (N2 Bristol strain, *N* = 911) and *Pristionchus pacificus* (PS312 strain, *N* = 601) adults reared on *E. coli* OP50-spotted and *E. coli* HT115-spotted NGM plates into 0, 20, 40, 60, 80 and 100% GSL water (diluted with ddH_2_O) for five minutes, transferred each individual back onto standard NGM plates, and assessed viability both five minutes and 24 h thereafter. We reared *P. pacificus* and *C. elegans* on one (*N*_1_ = 338) and three (*N*_1_ = 1150, *N*_2_ = 112, *N*_3_ = 211) different bacterial cultures from the microbialites of the GSL, respectively. These cultures came from three different microbialite samples collected from site 6, crushed and cultivated on NGM plates. We also isolated 124 nematodes from freshwater sediment (morphologically similar to nematodes recovered from freshwater input sites 1 and 2) collected from a wildlife refuge in Farmington Bay (salinity 0.01%) and determined their viability on standard NGM plates. Similarly, we placed *C. elegans* (*N* = 51) and *P. pacificus* (*N* = 30) in Farmington Bay water, and GSL nematodes (*N* = 36) collected from GSL State Park (40.74° N, 112.21° W) onto standard NGM plates, and assessed viability. For all survival assays, plates were incubated at 20°C and surviving animals were counted after five minutes and 24 h by their physiology and pick touch-provoked movement. Animals were recorded as dead if they met at least two of the following criteria: lack of spontaneous or touch-evoked swimming, grey discoloration, tissue degradation, body wall rupturing and stiff, especially straight posture [18].

### (f) Statistics

Nematode abundance across samples was log10(*n* + 1) transformed. Univariate ANCOVAs were performed using site as the categorical variable and salinity as a covariate. An ANOVA was performed using season within 2021 (spring, summer, autumn) as the categorical variable. Mann–Whitney Wilcoxon tests were performed on the subset of samples collected from GSL using microbialite status (microbialite versus non-microbialite) and year (2021 versus 2022) as a categorical variable. Univariate ANCOVAs were performed on the subset of samples collected from GSL using microbialite status as the categorical variable and pH, soil moisture content, or oxidation reduction potential as a covariate. For survival analyses, data for each individual (1, survived; 0, died) were encoded using generalized linear models with an underlying binomial distribution and a logit link function. For analysis of chemical environment, we performed Mann–Whitney Wilcoxon tests on the subset of samples collected from GSL using microbialite status as a categorical variable to see if there was a microbialite effect on the chemical environment. We also performed Mann–Whitney Wilcoxon tests on data from all sites to consider the GSL effect on each component of the chemical environment. Univariate ANCOVAs were performed using season as the categorical variable and salinity as a covariate on each component of the chemical environment. ANOVAs were performed to see if chemical environment influenced nematode abundance. Finally, we used the library {factoextra} to perform a principal component analysis on all numeric variables in our dataset. We also ran linear mixed model analyses with sample encoded as a random variable, which supported our interpretations of ANCOVAs and ANOVAs or indicated that the random effect was insignificant. All statistical tests were carried out in the R statistical environment (version 4.1.2, R Development Core Team 2019, http://www.r-project.org) in RStudio (version 1.4.869, RStudio Team 2019).

## Data Availability

Raw reads for 16S amplicon data have been deposited in the NCBI Sequence Read Archive (SRA) under BioProject number PRJNA927656. All data, including 18S sequences, and code to recreate analyses are publicly available through Dryad (https://doi.org/10.5061/dryad.nzs7h44wg) [63]. Supplementary material is available online [64].
